# Post-Translational Modifications of cGAS-STING: A Critical Switch for Immune Regulation

**DOI:** 10.3390/cells11193043

**Published:** 2022-09-28

**Authors:** Yang Yu, Jingyang Liu, Cun Liu, Ruijuan Liu, Lijuan Liu, Zhenhai Yu, Jing Zhuang, Changgang Sun

**Affiliations:** 1 College of Traditional Chinese Medicine, Shandong University of Traditional Chinese Medicine, Jinan 250355, China; 2 College of First Clinical Medicine, Shandong University of Traditional Chinese Medicine, Jinan 250355, China; 3 College of Traditional Chinese Medicine, Weifang Medical University, Weifang 261053, China; 4 Department of Oncology, Weifang Traditional Chinese Hospital, Weifang 261041, China; 5 Department of Special Medicine, School of Basic Medicine, Qingdao University, Qingdao 266071, China; 6 Department of Reproductive Medicine, Affiliated Hospital of Weifang Medical University, Weifang 261053, China

**Keywords:** cGAS-STING, post-translational modification, innate immunity, type I interferons, dsDNA sensing

## Abstract

Innate immune mechanisms initiate immune responses via pattern-recognition receptors (PRRs). Cyclic GMP-AMP synthase (cGAS), a member of the PRRs, senses diverse pathogenic or endogenous DNA and activates innate immune signaling pathways, including the expression of stimulator of interferon genes (STING), type I interferon, and other inflammatory cytokines, which, in turn, instructs the adaptive immune response development. This groundbreaking discovery has rapidly advanced research on host defense, cancer biology, and autoimmune disorders. Since cGAS/STING has enormous potential in eliciting an innate immune response, understanding its functional regulation is critical. As the most widespread and efficient regulatory mode of the cGAS-STING pathway, post-translational modifications (PTMs), such as the covalent linkage of functional groups to amino acid chains, are generally considered a regulatory mechanism for protein destruction or renewal. In this review, we discuss cGAS-STING signaling transduction and its mechanism in related diseases and focus on the current different regulatory modalities of PTMs in the control of the cGAS-STING-triggered innate immune and inflammatory responses.

## 1. Introduction

**Figure 1:**
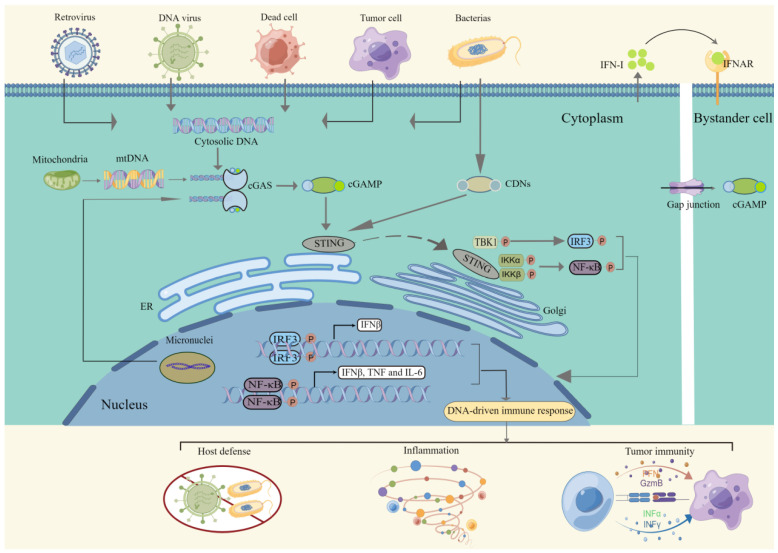
**The cGAS-STING-TBK1 signaling pathway.** Various DNA derived from virus, dying tumor cells or nucleus and mitochondria binds to and activates the cytosolic DNA sensor cGAS. cGAS utilizes GTP and ATP to produce the second messenger cGAMP, which directly binds to the ER-localized adaptor protein STING. Stimuli other than cGAMP, CDNs derived from bacteria can also activate STING. Subsequently, STING alters into a more closed conformation and transfers to the Golgi via the ERGIC, where it binds and activates the kinase TBK1 and IKK, which phosphorylate IRF3 and IκBα, respectively. Then, Phosphorylated IRF3 and IκBα dimerize and enter the nucleus, initiating the transcription of Type I IFN, TNF and IL6. These cytokines play a pivotal role in host defense, inflammation and antitumor immunity. Abbreviations: cGAS, cyclic GMP-AMP synthase; STING, stimulator of interferon genes; TBK1, TANK-binding kinase 1; cGAMP, cyclic GMP-AMP; ER, endoplasmic reticulum; CDNs, cyclic dinucleotides; ERGIC, ER-Golgi intermediate compartment; IKK, IκB kinase; IRF3, interferon regulatory factor 3; TNF, tumour necrosis factor.

Cell inherent recognition and defense systems against foreign genetic material encompass an ancient and fundamental feature of living systems. The innate immune response provides a critical first line of defense for host immunity via dynamic and complex interactions among its numerous cellular and molecular components. This gifted trait relies on germline-encoded pattern-recognition receptors (PRRs), which recognize microbial products, trigger signaling pathways, and produce soluble mediators, such as type I interferons (IFNs) and pro-inflammatory cytokines [1]. Type I IFN promotes cellular autonomous defense mechanisms in autocrine and paracrine manners by inducing the expression of IFN-stimulated genes, which in turn instructs and enhances the activation of the adaptive immune system [2]. Cytosolic DNA is a potent activator of the type I IFN response [3,4]. Cyclic GMP-AMP synthase (cGAS), an important cytosolic DNA sensor, binds DNA and activates stimulator of interferon genes (STING), which generates a signaling cascade, leading to the production of type I IFNs and other immune mediators [5,6] (Figure 1). Despite the multiple functions engaged by the cGAS-STING pathway in response to a variety of danger signals, dysregulated or sustained inflammation leads to pathological conditions, such as chronic infection and inflammatory autoimmune diseases [7]. Therefore, to coordinate the numerous cellular processes, the balance between the positive and negative regulation of the innate immune response triggered by cGAS-STING needs to be monitored. Post-translational modifications (PTMs), specifically phosphorylation, ubiquitination, SUMOylation, acetylation, methylation, and glutamylation, which induce covalent linkage to new functional groups greatly influence the activity and function of cGAS-STING-related proteins, thereby dynamically regulating immune homeostasis [8].

As an in-depth description of the structural and mechanistic insights of cGAS and STING has been reviewed in detail elsewhere, we only provide a concise summary in this review to clarify the object of our discussion. Here, we comprehensively reviewed the diversity and functionality of cGAS-STING PTMs in innate immune responses and highlighted their potential for clinical drug development.

## 2. Signaling of the cGAS-STING Pathway

In 2013, Li et al. first identified cGAS as a DNA receptor of the cytoplasmic DNA immune recognition pathway [9], after which the cGAS-STING signaling pathway became a significant research topic in the field of immunology. The cGAS-STING signaling pathway is a complex process involving three main stages: detection of dsDNA, intracellular signal transduction, and immune response activation.

### 2.1. Detection of dsDNA

cGAS, a member of the nucleotidyltransferase (NTase) family, possesses a flexible and poorly conserved N-terminal domain and a highly conserved C-terminal catalytic domain composed of an NTase core and Mab21 domains [10,11]. As an innate immune sensor, it detects various double-stranded DNA (dsDNA), including DNA derived from viruses, bacteria, mitochondria, micronuclei, and retroelements, which can be broadly categorized as either pathogenic or endogenous DNA. Notably, cGAS binds to dsDNA through contact recognition of the sugar-phosphate backbone regardless of the nucleotide sequence [12]. cGAS efficiently recognizes dsDNA with a minimum length of >40 bp [13,14], making it capable of responding to multiple danger signals, such as a vast number of microbial infections and self-dsDNA leakage resulting from cellular malfunction, which is common in precancerous cells [15,16].

cGAS activation requires cytosolic DNA; therefore, in the absence of DNA, cGAS is self-inhibited [11,17]. When bound to dsDNA, the cGAS dimer exposes the catalytic site, allowing the positively charged surface and its zinc finger to interact with the sugar-phosphate backbone of DNA. A subsequent induction of significant conformational changes of cGAS in the NTase domain results in a catalytic pocket for structural switch rearrangement, thus allowing ATP and GTP as substrates to catalyze the synthesis of 2′,3′-cyclic GMP-AMP (2′,3′-cGAMP) [18]. In addition, when cells undergo various stresses, such as oxidation, metabolism, or DNA damage, they can release Mn2+ from their organelles [19]. Related studies have demonstrated that cGAS activated by Mn2+ alone can synthesize 2′3′ -cGAMP more efficiently [20,21].

### 2.2. Intracellular Signal Transduction

2′,3′-cGAMP, a cyclic dinucleotide (CDN), binds to and activates the transmembrane receptor protein STING as a second messenger. Additionally, cGAMP contains a unique hybrid phosphodiester bond that can be transferred from one cell to another through intercellular gap junctions, thereby amplifying STING signaling across neighboring cells [22,23,24].

STING is an endoplasmic reticulum (ER) membrane-bound protein composed of four transmembrane domains: a CDN-binding domain (CBD) and a C-terminal tail (CTT) with binding sites for TANK-binding kinase 1 (TBK1) and IFN regulatory factor 3 (IRF3). Binding of 2′,3′-cGAMP to the STING dimer on the ER leads to a conformational change, forming STING tetramers and high-order oligomers [25,26]. These conformational changes induce translocation of STING to the ER–Golgi intermediate compartment (ERGIC) and Golgi apparatus in a process that is dependent on complex II (COPII) complex and ADP-ribosylation factor (ARF) GTPases [27,28]. During this process, STING interacts with TBK1 and promotes autophosphorylation of TBK1 [29]. In turn, the CTT region of STING is phosphorylated by TBK1 as well. Phosphorylated STING binds to the positively charged region of IRF3, resulting in IRF3 phosphorylation and dimerization via TBK1 [30,31]. The active IRF3 dimer translocates to the nucleus and activates the transcription of type I IFN genes and IFN-stimulated genes (ISGs) [32,33], ultimately augmenting a fast, robust innate immune system and the resulting adaptive immunity. In addition, STING acts as an adaptor protein for the Ku 70 protein DNA sensor, activating IRF1 and IRF7, and inducing the production of IFN-λ1 (type III IFN), which has been described in various cell lines [34,35,36]. STING can also bind to and stimulate IκB kinase (IKK)-mediated nuclear factor-κB (NF-κB)-driven inflammatory gene production [37]. Upon signal transduction termination, STING is transferred to endolysosomes and degraded [38].

### 2.3. Immune Response Activation

Oligomers formed by STING activation trigger pleiotropic downstream events, of which the activation of IRF3 and NF-κB-dependent signaling cascade responses are the most representative hallmarks. TBK1 and STING co-phosphorylate IRF3. Subsequently, dimerized IRF3 and phosphorylated NF-κB enter the nucleus to jointly drive the expression of downstream type I IFNs, pro-inflammatory cytokines, and chemokines [39]. As immunomodulators, IFNs can selectively increase antigen cross-presentation and regulate T-lymphocyte differentiation, thus linking innate and adaptive immunity and enhancing a more intense immune response [40,41].

## 3. Regulatory Mechanisms of cGAS-STING in the Context of Disease

### 3.1. Viral and Bacterial Infections

Innate immunity is the host’s first line of defense against viral invasion and is critical for eliciting the subsequent adaptive immunity to ultimately eradicate an infection [42]. The human immune system senses pathogenic infections through PRRs, a critical “life or death” event that governs virus emergence in host populations. Subsequently, defense mechanisms against viral infection trigger a series of cascade events that eventually result in the transcriptional activation of type I IFN and pro-inflammatory cytokines. Type I IFNs are known to either directly restrict viral replication or induce host cells to inhibit viral protein synthesis [43]. A large body of evidence clearly demonstrates that the cGAS-cGAMP-STING pathway has emerged as a critical defense mechanism in antiviral responses, partly caused by cytoplasmic DNA recognition and type I IFN production [44,45]. Notably, infection with many different types of viruses, including DNA viruses, lentiviruses, and RNA viruses, can trigger cGAS-STING signaling [46,47,48,49]. The regulatory mechanisms between these viruses and their hosts are summarized in Table 1.

Evidently, cGAS was initially characterized as a classical DNA-binding protein that plays a crucial defensive role in the response of type I IFNs to DNA viruses [68,78]. In addition to cGAS-DNA interactions, many studies have highlighted the importance of cGAS-STING in RNA virus biology and disease pathogenesis, although previous studies have focused on RNA sensors against RNA viruses. It is essential to mention that this potential role does not imply that cGAS can recognize viral RNA or directly activate RNA interactors. The detailed mechanism of cGAS in the detection of RNA viral genomes is not well understood. Recent studies have reported the RNA-binding activity of cGAS, which could largely modulate rather than activate it [152], and more evidence is needed to confirm this. Different RNA viruses may activate the cGAS-STING pathway through multiple pathways. For example, cytoplasmic mtDNA induces leakage of damaged mitochondria, fusion sensing of enveloped viruses [146,153], or self-DNA release into the cytoplasm because of damage to the nuclear envelope [148]. In addition, a recent study has shown that STING can restrict the replication of various RNA viruses at the translational level, which will be described later [143]. By rapidly initiating a type I IFN-dependent immune response and limiting viral replication, the cGAS-STING signaling axis has emerged as a pathway with the potential to treat multiple RNA viruses. To realize this potential, a more detailed elucidation of the actual involvement of cGAS and STING in RNA viral infections is urgently needed. Surprisingly, several RNA virus families have been shown to replicate productively through the non-classical functions of the cGAS-STING pathway, such as autophagic degradation, lipid recognition and T cell responses [36,68,115,154]. Given the evolutionary pressure on viruses, the emergence of a growing number of new schemes demonstrates the importance and complicated network manipulation of cGAS-STING signaling cascades during viral escape, presenting a cat-rat race for survival between viruses and their hosts.

Furthermore, the cGAS-STING pathway is widely involved in various bacterial infections. cGAS and STING detect bacterial genomic DNA and bacterial cyclic dinucleotides (CDNs) present in the cytoplasm, respectively, and thus affect the outcome of intracellular bacterial infection [155,156,157]. CDNs released by many bacteria, such as Listeria monocytogenes [158], Neisseria gonorrhoeae [159], Mycobacteria, Legionella, Shigella, Francisella, group B streptococcus, and Chlamydia [16], are potent stimulators of type I IFN induction as well and are sensed by IFI16 and cGAS to activate the STING pathway [160,161]. Notably, unlike the protective effects against viruses, type I IFN plays a protective or deleterious role in the host depending on the bacterial species and infection mode [157]. Thus, the cGAS-STING signaling pathway is more complex and diverse in bacterial infections. Future studies will further clarify the regulatory rules of cGAS-STING in various bacterial infections.

### 3.2. Cancer

The extensive study of cGAS as a key immune regulation engine during viral invasion and bacterial infection has vastly increased our understanding of innate immunity. The cGAS-STING pathway challenges the traditional pathogen-specific structural model and may ensure a correct immune response through different regulatory mechanisms. Whether this new perspective can participate in the cancer-induced immune effect deserves further research, especially with the increase of tumor immunotherapy, which has become particularly important.

There is an innate advantage of the cancer-activated cGAS-STING pathway, for cancer cells share common characteristics, including genomic instability, mutations or deletions of oncogenes, oxidative stress, and exuberant metabolism [162,163]. In these stressed states, micronucleus and mtDNA are prone to leakage [164]. This unstable dsDNA subsequently initiates the cGAS-sensing cascade and hinders early neoplastic cell progression [165]. Exogenous stimuli such as exposure to ionizing radiation or treatment with chemotherapeutic agents can likewise induce DNA damage, which participates in the accumulation of cytoplasmic DNA in tumor cells and acts as a promoter to induce STING-dependent type I IFN [166,167]. Specifically, the activation of cGAMP can also directly trigger tumor immunity, resulting in robust tumor clearance [168]. Intratumoral injection of 2′,3′-cGAMP significantly retarded tumor growth and reduced lung metastasis in B16F10 mouse models [169]. Furthermore, nuclear cGAS has been shown to promote lethal accumulation of DNA damage in the cancer genome by inhibiting homologous recombination (HR) DNA repair [170].

I. Enhanced adaptive immunity 

In the tumor microenvironment, the immune surveillance mechanism of cGAS-STING has been well characterized and is mediated by infiltrating immune cells, such as dendric cells (DCs) and T cells, through type I IFN signaling [171]. Researchers found that tumor-derived DNA and cGAMP activate the cGAS-STING pathway in DCs via an unknown mechanism, which in turn performs cross-presentation to DCs and recruits CD8+ T cells for direct, non-spontaneous tumor elimination [170,172]. In addition, type I IFN drives the production of multiple chemokines, such as CXCL9 and CXCL10, which are required for cytotoxic T lymphocyte (CTL) metastasis and infiltration [173]. Accordingly, the regulatory functions of CD8+ T cell activation and differentiation provide significant anti-tumor effects in the STING pathway.

A recent study found that type I IFN in this pathway also interferes with immunosuppression of regulatory T (Treg) cells by downregulating phosphodiesterase 4 (PDE4) and upregulating cyclic adenosine monophosphate (CAMP) [174]. Moreover, in response to various changes and stimuli in the intracellular environment, the cGAS-STING signaling pathway promotes the polarization of tumor-associated macrophages toward the M1 phenotype, thereby secreting inflammatory factors and chemokines to recruit and activate T lymphocytes [175]. Xu et al. further verified through in vitro experiments that STING may regulate the phenotype and anti-tumor effects of macrophages through the IL6R-Jak-STAT-IL24 signaling pathway [176].

Activation of STING in non-malignant cells also has tumor-suppressive effects. Under appropriate conditions, STING signaling can indirectly inhibit melanoma growth by supporting the spontaneous response of natural killer (NK) cells to tumor cells [116,177]. Further studies have shown that the anti-tumor immune function of NK cells depends largely on STING activation in non-malignant cells, which is considered an important component of innate immunity and may serve as a target for immunotherapy of related diseases [177].

II. Senescence and Cell Death

Multiple studies have provided strong evidence that cGAS is an important molecular link between DNA damage, senescence-associated secretory phenotype (SASP) gene expression, and aging [178,179]. Tumor aging may denote a favorable outcome as an endogenous barrier to malignant transformation. cGAS recognizes cytosolic chromatin fragments in senescent cells and promotes the transcription of SASP via the STING pathway, which in turn prevents abnormal proliferation of damaged cells in autocrine and paracrine forms [180], particularly in precancerous stages [181]. Moreover, the differences in the application of the cGAS pathway-induced senescence in precancerous, early, and advanced cancers deserve further investigation.

Through transcriptional activation of apoptotic regulators and IRF3, the cGAS-STING signaling pathway initiates a cascade of responses that support anti-proliferative cell states, including necroptosis, apoptosis, and pyroptosis, which are involved in tumorigenesis and metastasis. The necrotrophic apoptotic capacity of STING is closely related to the induction of pro-apoptotic BH3-only proteins compared with those undergoing senescence [180,182]. Noxa and PUMA are involved in both in vivo and in vitro STING-mediated apoptotic processes [182,183]. Alternatively, in a more direct mode of action, phosphorylated IRF3 can combine with B-cell lymphoma 2 (Bcl-2) related X protein (BAX) and Bcl-2 homologous antagonist/killer (BAK) to form a complex that directly drives downstream non-transcription-dependent apoptosis induction [184,185]. Additionally, STING interacts with splenic tyrosine kinase (Syk) and regulates pyroptosis in colitis-associated colorectal cancer [186].

cGAS/STING also promoted more immediate anti-tumor effects. Cellular autophagy is the key effector activity that drives a robust autophagic cell death program and exerts synergistic anti-tumor effects. Interestingly, in evolutionary terms, autophagy is a primitive function of STING and may have preceded the role of STING in IFN signaling [187]. Chen et al. revealed that these two pathways were completely independent [27]. Despite this, an accurate understanding of the molecular mechanisms of the STING-autophagy–cell death axis is still lacking. The underlying pathways may be involved in the regulation of the ER stress response [188], mitochondrial autophagy [189], or calcium signaling [190].

### 3.3. Autoimmune Diseases

The versatility of the cGAS-STING pathway enables it to play a critical role in detecting and eliminating multiple threats. However, its potency is a double-edged sword, with under-activation leading to an inadequate response to threats, such as cancer or viruses, and its over-activation is associated with the development of inflammatory and autoimmune diseases, such as systemic lupus erythematosus (SLE) [191], Aicardi–Goutières syndrome (AGS), STING-associated vasculopathy with onset in infancy (SAVI) [192], and neurodegenerative diseases [193]. 

cGAS senses dsDNA regardless of its origin and is therefore unable to distinguish between endogenous and foreign DNA. Deoxyribonucleases (DNases) prevent endogenous DNA accumulation by degrading self-DNA in different compartments under normal physiological conditions. Unfortunately, loss-of-function mutations in DNA exonuclease three-prime repair exonuclease 1 (TREX1) interfere with DNA degradation in apoptotic cells and lead to excessive type I IFN signatures that ultimately develop into SLE or AGS [194,195]. Previous studies have shown that TREX1-deficient mice develop inflammatory myocarditis, lymphoid hyperplasia, vasculitis, and kidney disease [196], which can be fully rescued by knockout of the cGAS gene [197]. Similarly, DNase II is a lysosomal endonuclease responsible for the degradation of DNA from engulfed apoptotic cells. DNase II-deficient mice die intra-embryonically owing to the development of anemia and overproduction of type I IFNs [198]. Intriguingly, further deletion of STING completely rescued embryonic lethality and the autoimmune and inflammatory phenotypes [197]. Furthermore, gain-of-function mutations in STING have been proven to be pathological mediators of the auto-inflammatory diseases, such as SAVI [199,200]. Collectively, these human and mouse genetic studies provide conceptual clues for targeting the cGAS-STING pathway for the treatment of certain human inflammatory diseases.

Self-released DNA that leaks from the nucleus or mitochondria can also function as a cGAS ligand to activate the pathway and cause severe inflammatory responses. A previous study found that self-DNA from apoptotic cells activates cGAS, which is the leading cause of myocardial infarction-associated type I IFN production [201]. In addition to damaged nuclear DNA, cGAS also responds to other self-DNA that is mislocalized to the cytoplasm and drives self-inflammation. In Parkinson’s disease, mtDNA dysregulation has been noted, which may also be implicated in cGAS binding and type I IFN induction [193]. Notably, intercellular transfer of cGAMP appears to exacerbate inflammation. A mouse model of alcohol-induced liver disease showed that the transfer of cGAMP as an extracellular immune transmitter increases inflammation and disease severity [202]. Given the complexity of the cGAS-cGAMP-STING signaling process, the role of cGAMP transfer in disease severity remains an area worthy of future exploration.

## 4. PTM Networks Regulating the cGAS-STING Pathway

**Figure 2:**
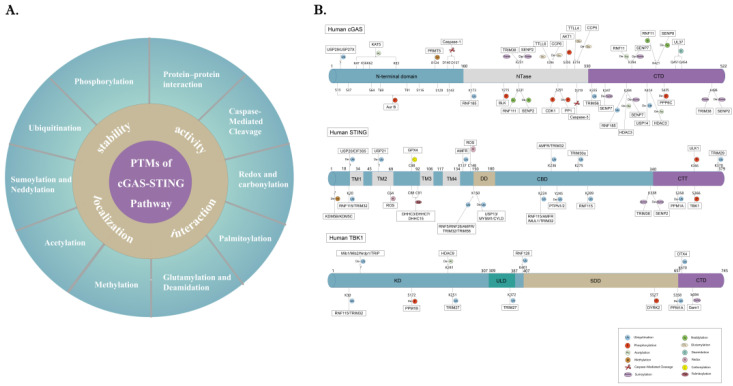
**Overview of PTMs of cGAS-STING pathway.** (**A**) PTMs play a critical role in regulating cGAS-STING pathway, including protein stability, activity, localization, and interaction. (**B**) Defined modification sites for cGAS/STING/TBK1 (ubiquitination, phosphorylation, acetylation, methylation, caspase-mediated Cleavage, sumoylation, neddylation, glutamylation, deamidation, redox, carbonylation, and palmitoylation) and their modifying enzymes are plotted. Different colors are used to differentiate distinct modification types. Abbreviations: NTase, nucleotidyl transferase; CTD, C-terminal domain; TM, transmembrane domain; DD, dimerization domain; CBD, cyclic dinucleotide-binding domain; CTT, C-terminal tail; KD, kinase domain; ULD, ubiquitin-like domain; SDD, scaffold and dimerization domain.

The fine regulatory patterns of innate sensors and downstream signaling molecules are of vital importance in cell fate decisions [203]. As a critical regulatory event, PTMs play an important role in regulating protein activity, localization, stability, and protein–protein interactions by inducing their covalent linkage to new functional groups, such as phosphates, methyl, and acetate [8]. The complex structure of cGAS/STING makes it a perfect platform for a multitude of covalent modifications, including phosphorylation, ubiquitination, acetylation, methylation, and SUMOylation (Figure 2). These PTM fine regulatory networks generate a robust yet “tunable” cytokine response that contributes to sustaining immune homeostasis in distinct contexts.

### 4.1. Phosphorylation

Phosphorylation is the most extensively investigated PTM in innate immunity and is inversely regulated by protein kinases and phosphatases. Specifically, protein phosphorylation refers to the addition of phosphate groups to tyrosine, threonine, or serine under the catalysis of phosphorylating kinases. Studies have shown that complex enzyme phosphorylation networks are widely used in signaling pathways as the preferred modality for innate immune regulation in vivo [204].

In the cGAS-STING pathway, cGAS (de)phosphorylation has been shown to be essential for regulating cGAS subcellular localization or activity. AKT serine/threonine kinase 1 (AKT1), also known as protein kinase B (PKB), regulates many physiological processes including proliferation, metabolism, growth, and cell survival. Seo et al. demonstrated that cGAS is a substrate for the serine/threonine protein kinase AKT, which mediates phosphorylation at S291 (mouse) or S305 (human), and suppresses the enzymatic activity of cGAS, thereby leading to a negative immune response in the host. In contrast, the AKT inhibitor VIII and the S291A mutant of cGAS promoted DNA-induced IFN-β production and inhibited herpes simplex virus (HSV)-1 infection [205]. Unlike AKT, the mitotic kinase cyclin-dependent kinase 1 (CDK1)-cyclin B complex only phosphorylates the S291 site of mouse cGAS during intermittent mitosis, inhibiting cGAMP synthesis. In particular, when cells exit mitosis, this phosphorylation can be reversed by protein phosphatase 1 (PP1), thereby restoring their DNA-sensing capacity [206]. Another study showed that during mitosis, the N-terminus of cGAS is phosphorylated by mitotic kinases, including Aurora kinase B, which then block chromatin DNA sensing [207]. Oligomerization of chromatin-bound cGAS is inhibited, which is required for cGAS activation. Together, these mechanisms ensure that cGAS is inactive when bound to chromatin during mitosis, potentially contributing to the prevention of autoimmune responses. In murine experiments, Shu et al. reported that another protein serine/threonine phosphatase (PSP) family member, PPP6C, inhibited phosphorylation of mouse S420 (human S435), thereby preventing its binding to GTP and inhibiting cGAMP synthesis [208]. In addition to serine phosphorylation, cGAS is also influenced by tyrosine phosphorylation, which regulates its subcellular localization. B-lymphoid tyrosine kinase promotes cGAS phosphorylation at Y215 in resting cells to control the cytosolic retention [170].

Serine phosphorylation events are also essential for both STING and IRF3 activation. The phosphorylation of STING S358 allows TBK1 to bind STING. Then, TBK1 trans-phosphorylates S366 of the adjacent STING complex, allowing the recruitment, phosphorylation, and dimerization of IRF3 [29,30,31]. Conversely, Konnon et al. reported that phosphorylation of activated STING on identical residues by the autophagy-related serine/threonine protein kinases ULK1 and ULK2 increases STING degradation without IRF3 activation [209]. As can be seen, these apparently contradictory results require further investigation to reconcile the discrepancies. Consistent with this, protein phosphatase Mg2+/Mn2+- dependent 1A (PPM1A) has been reported to target and dephosphorylate STING at S358 [209,210]. This inverse inhibitory phosphorylation plays a positive role in attenuating and terminating the type I IFN signaling pathway in the later stages of DNA virus infection. Additionally, ribosomal protein S6 kinase 1 (S6K1) has also shown to be involved in STING phosphorylation. S6K1 interacts with TBK1 to form a ternary signaling complex that promotes signal transduction along the cGAS-STING-TBK1-IRF3 axis [211]. Subsequent experiments revealed that the phosphorylation of tyrosine residues also contributes to the regulation of STING. Tyrosine-protein phosphatase non-receptor type (PTPN) 1 and 2, which act as MITA/STING-related proteins, mediate MITA/STING Y245 dephosphorylation to facilitate STING degradation by the 20S proteasome and attenuate the intrinsic antiviral response [212].

The kinase activity of TBK1 is critical downstream of STING. Numerous serine (S)/threonine (T) sites on the TBK1 protein can be phosphorylated, acting as negative regulators of risk factor-triggered type I IFN induction. Dual-specificity tyrosine-(Y)-phosphorylation-regulated kinase 2 (DYRK2) induces TBK1 S527 phosphorylation and K48-linked ubiquitination in a kinase activity-dependent manner [213]. Furthermore, PPM1B was identified as a TBK1 phosphatase that removes TBK1 autophosphorylation to eliminate downstream IRF3 activation and negatively regulate antiviral responses [214]. Several studies have reported that IRF3 is regulated via phosphorylation modifications. DNA-dependent protein kinase (DNA-PK) functions as a potent regulator of the IRF3 pathway and can phosphorylate IRF3 at Thr135, leading to its retention in the nucleus and delay in its proteolysis process [215]. In line with this, PTEN releases IRF3 and promotes its nuclear translocation by reversing phosphorylation of the IRF3 S97 site [216]. In contrast, protein phosphatase 2A (PP2A) mediates IRF3 dephosphorylation, resulting in the negative regulation of type I IFN production [217]. How phosphorylation and dephosphorylation of IRF3 are precisely balanced to ensure the appropriate type I IFN production remains to be fully resolved.

### 4.2. Ubiquitination

Ubiquitination is an essential PTM that involves covalent attachment of ubiquitin (Ub) molecules to a target protein. The ubiquitination system is dependent on a sequential enzymatic cascade of E1 (Ub-activating enzyme), E2 (Ub-conjugating enzyme), and E3 (Ub ligase). Furthermore, these cellular events can be counteracted by Ub hydrolases/deubiquitinases (DUBs) by removing Ub from the substrate protein. Taken together, the dynamic conversion between ubiquitination and deubiquitination plays a crucial role in the maintenance of cellular homeostasis [218].

Regulation of protein ubiquitination is most commonly dependent on E3 ligase activity. cGAS ubiquitination is also inversely modified by several E3 enzymes or DUBs, and plays critical roles in a wide variety of immunological processes, such as antigen processing. Among them, E3 Ub ligases, RNF185 and TRAF6 are responsible for the polyubiquitination of cGAS, while TRIM56 and TRIM41 monoubiquitinate cGAS. RNF185 specifically catalyzes the formation of the K27-linked polyubiquitin chain at K173 and K384 of cGAS, promoting its enzymatic activity [219]. TRAF6, another E3 ubiquitin ligase, also promotes the polyubiquitination of cGAS to upregulate cGAS-dependent type I IFN signaling [220]. In contrast to TRAF6, TRIM56 induced cGAS monoubiquitination at K335 of the CTD structural domain, leading to a significant increase in cGAS dimerization, DNA-binding activity, and cGAMP production. Congruently, one study suggested that macrophages from Trim56−/− mice produced significantly less IFN-β mRNA when infected with HSV-1 instead with influenza virus [149]. Similar to TRIM56, TRIM41, another TRIM family E3 ubiquitin ligase, is responsible for monoubiquitinating cGAS and upregulating cGAS activity as well as cGAMP synthesis [221]. In addition, cGAS K414 is constitutively K48-linked ubiquitinated and then degraded via the p62-mediated autophagy-lysosomal pathway rather than via the ubiquitin–proteasome system. In this study, the researchers also observed that TRIM14 promotes cGAS enzymatic activity upon viral infection, while recruiting the deubiquitinase USP14. USP14 cleaved the K48-linked polyubiquitin chain of cGAS, thus disrupting the cGAS-p62 interaction and preventing the autophagic degradation of cGAS [222]. Both the deubiquitinating enzymes, USP27x and USP29, interact with cGAS in a similar way and mediate the removal of the K48-linked polyubiquitinated chain to stabilize cGAS [223,224].

To positively regulate STING function, the E3 ubiquitin ligases TRIM56, TRIM32, RNF115, and MUL1 facilitate STING dimerization and its interaction with TBK1 by promoting the conjugation of K-63 linked chains, which are two critical steps in STING-mediated signaling. Specifically, TRIM32 and TRIM56 jointly target STING at K150 for K63-linked polyubiquitination, thereby enhancing the activation of downstream pathways [225,226]. The exact mechanism by which TRIM32 and TRIM56 coordinate STING in this process remains to be elucidated. Furthermore, the mitochondrial E3 ubiquitin protein ligase 1 (MUL1) and RNF115 are responsible for the K63-linked polyubiquitination of STING at K224 and K20, K224, and K289 sites, respectively, to boost signaling from TBK1 to IRF3 [227,228]. Intriguingly, MUL1-mediated ubiquitination of STING is required for STING-IRF3 activation, whereas STING-NF-κB signaling does not [227,228]. Contrary to this, to maintain cellular homeostasis and immune response, cytoplasmic DNA stimulation induces the expression of Myb-like, SWIRM, and MPN domains 1 (MYSM1) protein, which interacts with STING and cleaves STING K63-linked ubiquitination at K150 to limit STING over-activation [229]. Furthermore, the autocrine motility factor receptor (AMFR) interacts with STING and catalyzes K27-linked polyubiquitination at multiple sites (K137, K150, K224, and K236), thereby positively regulating STING-dependent signaling activation. Specifically, the interaction between AMFR and STING is enhanced by another protein, INSIG1, to promote downstream signaling [230]. However, a recent study indicated that the deubiquitinating enzyme USP13 could reverse this modification and prevent TBK1 from recruiting signaling complexes, thereby impairing cellular antiviral responses [231]. The same is true for another deubiquitinating enzyme, USP21 [232]. K63-linked polyubiquitination is involved in signal transduction, whereas K48-linked polyubiquitination is involved in proteasomal degradation. TRIM30α and TRIM29 induce termination of signaling activation by K48-linked polyubiquitination of STING at K275 and K370, respectively, and subsequent degradation [233,234]. A subsequent experiment showed that knockdown and deficiency of TRIM30α resulted in high levels of type I IFN and IL-6 production upon dsDNA stimulation, and mice with TRIM30α-/- were more resistant to HSV-1 infection than wild type mice [235]. Notably, TRIM30α is not present in humans [236]. Similarly, E3 ubiquitin ligases RNF5 and RNF90 were identified as STING-interacting molecules capable of K48-linked ubiquitination at K150 during viral infection, leading to degradation of its proteasome [237,238]. RNF26, in contrast, adds a K11-linked ubiquitin chain at the same lysine to induce STING ubiquitination, which prevents STING from RNF5-mediated premature STING degradation and enhances innate immune signaling [239]. Notably, inactive rhomboid protein 2 (iRhom2) can also recruit the deubiquitinating enzyme EIF3S5 to stabilize STING by removing the K48-linked polyubiquitin chain mediated by RNF5 [240]. Moreover, studies on iRhom2 have furthered our understanding of the intracellular trafficking in STING signal transduction. The important translocation-associated protein, TRAPβ, is activated by iRhom2, which may facilitate STING transport from the ER to the perinuclear membrane region [240]. However, HCMV tegument protein UL82 acts as a negative regulator and interferes with the STING pathway by disrupting the iRhom2-mediated STING-TRAPβ translocation complex [89]. Recently, USP13 was also shown to function as a deconjugating enzyme for K27-linked and K33-linked polyubiquitination of STING, although the exact mechanism is not known yet [231]. Two other ubiquitin-specific peptidases, USP18 and USP20, were also found to prevent STING degradation following DNA viral infection [241]. Moreover, CYLD, a deubiquitinase, can stabilize STING protein function by deconjugating K48-linked polyubiquitination, contributing to the positive regulation of the natural immune response [242].

Given the importance of STING signaling for host antiviral responses, many viral proteins have evolved to manipulate STING deubiquitination to evade the immune surveillance system. Both HSV-1 VP1-2 and HTLV-1 Tax inhibit the interaction between TBK1 and STING by causing its deubiquitination [58,243]. Likewise, Epstein–Barr virus (EBV) regulates the expression of TRIM29, which further promotes K48-linked polyubiquitination, thereby interfering with STING-mediated antiviral responses [234]. Analogously, Hepatitis B virus (HBV) has been shown to utilize ubiquitination modifications to negatively regulate STING activity and evade the antiviral type I IFN response [85,244]. Furthermore, innate immune sensing mediated by the STING pathway is also critical for eliciting antitumor immune responses against a wide range of cancers. A recent study identified death-associated protein kinase 3 (DAPK3) as a pivotal regulatory complex that drives tumor-intrinsic immunity and immune surveillance through STING-mediated ubiquitination and phosphorylation [245]. In particular, the detailed mechanisms of PTM crosstalk need to be explored in the future.

Interestingly, in addition to STING, TBK1 is another important target for ubiquitination modification. Several E3 ubiquitin ligases, including Nrdp1, Mib1, Mib2, and RNF128, have been identified to induce K63-polyubiquitin conjugation of TBK1 residues under different stimuli to augment their activity [246]. Furthermore, some E3 ubiquitin ligases, including DTX4, TRIP, and TRIM27, specifically interact with TBK1 and trigger polyubiquitination of the K48 linkage at K251 and K372, causing the degradation of TBK1, thereby modulating its stability [247]. The multi-level modification of IRF3 has been a research hotspot in recent years, and the most common modification is ubiquitination involving K48. Moreover, E3 ubiquitin ligases RBCK1, RAUL, and Ro52 contribute to IRF3 proteasomal degradation and inhibit IRF3-dependent antiviral signaling by mediating the ubiquitination of IRF3 at K48 [248,249,250]. Through this modification, proline isomerase Pin1 has also been shown to affect innate signaling and antiviral defense [251].

### 4.3. SUMOylation and Neddylation

In addition to ubiquitination, another small ubiquitin-like modifier covalently linked to the target protein, small ubiquitin-like modifier (SUMO), has also been characterized. SUMOylation is regulated by specific SUMO E1, E2, and E3 cascades, and desulfurases. Similar to ubiquitination, SUMOylation and its reverse reaction, deSUMOylation, can respond rapidly to changing external and internal stimuli and orchestrate large biological immune effects through dynamic alterations in the biochemical properties of the substrate [252].

Members of the tripartite motif (TRIM) family of proteins are well-known for their roles as E3 ubiquitin ligases. Recently, several studies have demonstrated that TRIM molecules, in addition to triggering ubiquitination, are involved in the SUMOylation modification of cGAS-STING signaling. Early in the immune response, TRIM38 targets cGAS and STING to elicit a biochemical reaction of SUMOylation, thereby preventing K48-linked ubiquitination and degradation [253]. As a contrast, the sentrin-specific protease (SENP) family catalyzes the deSUMOylation process and regulates the dynamic equilibrium of the SUMO conjugation–deconjugation cycle during the later stages of viral infection. For instance, SENP2 removes SUMO modifications from cGAS and IRF3, thus avoiding excessive and potentially harmful immune responses [254,255]. In addition, these modifications were mapped to lysine residues 335, 372, and 382 of cGAS, impairing cGAS-DNA binding, oligomerization, and nucleotidyltransferase activity, ultimately weakening the innate antiviral response [253,256]. To maintain homeostasis of the immune environment, SENP7 relieves this inhibition by catalyzing de-SUMOization of cGAS [256]. Several studies have highlighted the critical role of TBK1-mediated SUMOization in antiviral responses. Mass spectrometry allowed the identification of TBK1 at K694 as a SUMO acceptor site, which could directly modulate the antiviral function of TBK1 by damping interactions with adaptor proteins [257]. Some viral proteins, such as the adenovirus early gene product Gam1, have been identified as substrates for SUMO modification, which can be exploited to intentionally manipulate the SUMO system in vivo or in vitro, thus affecting the antiviral functions [257]. Considering the contribution of TBK1 SUMOylation to its antiviral activity, it will be interesting to screen for more viral proteins interfering with its SUMOylation status in the future.

NEDD8, like ubiquitin and other ubiquitin-like proteins (UBLs), is added to the lysine residue of its target substrate by a three-step enzymatic cascade. Indeed, neddylation is also involved in the innate immune responses mediated by cGAS. Recent studies have proposed that the Ube2m-RNF111 axis, acting as an NEDD8 E2-conjugating enzyme and NEDD8 E3 ligase, is responsible for the neddylation of cGAS [258,259]. Consistently, mice deficient in Ube2m or RNF11 showed less resistance to HSV-1 infection [259]. Therefore, this positive PTM is vital for strengthening of cGAS dimerization and its dsDNA-binding ability. Nevertheless, the exact mechanism by which the neddylation pathway affects the functions and mechanisms of the cGAS-mediated DNA sensing immune pathway remains unclear.

### 4.4. Acetylation

Acetylation of histone lysine residues is a crucial epigenetic modification that affects histone structure and gene expression, and supports a number of cellular regulatory systems, such as regulation of protein function, chromatin structure, and signal transduction. The acetylation of cGAS has recently attracted much attention. To date, researchers have identified several cGAS de/acetylation sites (K21, K47, K50, K56, K62, K63, K82, K83, K198, K384, K392, K394, and K414) using mass spectrometry [260,261,262]. Among these sites, K47, K56, K62, and K83 of N-terminal structural domain of cGAS are targeted for acetylation by KAT5 to increase its DNA-binding ability and ultimately enhance cGAS immune signaling [260]. Furthermore, KAT2A belongs to another acetyltransferase family and directly exerts its effectiveness via regulating the acetylation level of cGAS, which promotes inflammation amplification in SLE [263]. Considering the complexity of PTM, the same PTM at different sites may result in different cGAS activity outcomes. For instance, acetylation of cGAS K384/K394/K414 may limit its activity by suppressing cGAMP synthesis, thus negatively affecting its role in immune signaling [262]. Moreover, acetylation of K384 and K414 hindered cGAS-dependent apoptosis [261]. Interestingly, under the stimulation of DNA, histone deacetylase 3 (HDAC-3) interacts with cGAS to catalyze its deacetylation and participates in cGAS activation [262,264]. Hence, these results suggest that both KAT-mediated acetylation and KDAC-mediated deacetylation activate cGAS and modulate the type I IFN response, albeit at different sites. Similarly, acetylation contributes to the regulation of cGAS activity. Another histone deacetylase, HDAC-9, directly deacetylates TBK1 and stimulates its kinase activity [265].

### 4.5. Methylation

Akin to acetylation, methylation is an important epigenetic marker of histone tails and is emerging as a potent regulator of innate immunity via epigenetic modulation of chromatin status and DNA transcription. Several studies have highlighted that some arginine and lysine residues in cGAS-STING can also be methylated. Han et al. reported the presence of cGAS methylation in HEK293T cells and its regulation by protein arginine methyltransferase 5 (PRMT5). Specifically, PRMT5 blocks cGAS-DNA binding capacity by inducing arginine symmetric dimethylation of cGAS at Arg124, and further attenuates cGAS-mediated antiviral innate immunity [266]. Thus, signaling initiation by arginine methylation allows the characterization of direct methylation modifications of the cGAS protein and defines PRMT5 as a negative regulator of cGAS-mediated type I IFN production during viral infection. Emerging evidence suggests that STING expression is also epigenetically suppressed by the histone H3K4 lysine demethylases KDM5B and KDM5C, which obstruct the signal transduction initiated by endogenous DNA and is mediated by the cGAS-STING-TBK1-IRF3 axis [81]. However, the specific regulatory mechanisms underlying such modifications remain to be elucidated.

### 4.6. Glutamylation and Deamidation

A distinct PTM, glutamylation, attaches glutamate side chains to glutamic acid residues in the primary sequence of the target proteins. Recently, glutamylation was identified as a critical regulator of cGAS function that has profound effects on DNA binding and activation [267]. For example, tubulin tyrosine ligase-like (TTLL) family members negatively regulate the inflammatory response triggered by cGAS through various mechanisms. TTLL6 primarily targets cGAS at E286 for polyglutamylation to impair its affinity for DNA, whereas TTLL4 promotes monoglutamylation of the E314 site to hinder its synthase activity [268]. On the contrary, the intracellular carboxypeptidases CCP5 and CCP6 can release the inhibitory effects of glutamylation and recover cGAS activity, suggesting that cGAS function is tightly controlled by cellular glutamylation and de-glutamylation [268]. Remarkably, in both cases, only a part of the cGAS protein was inhibited by glutamylation modification. Therefore, it is imperative to conduct further research to explain how this modification affects the functions of unmodified cGAS.

Deamidation has emerged as a feasible, effective, and attractive method of protein modification. Specifically, it refers to the process of deamidating the side chain of asparagine (Asn) or glutamine (Gln) residues to produce aspartic acid (Asp) or glutamic acid (Glu), respectively, under the catalysis of deamidase, which is involved in various biological functions [269]. Currently, the HSV UL37 protein is a known deamidase that deamidates the Mab21 enzymatic domain of cGAS. Specifically, it reduces the cGAMP-synthesizing activity of cGAS, thereby preventing double-stranded RNA (dsRNA)-induced innate immune activation [59]. Nevertheless, it remains unclear whether the deamidation of cGAS crosstalk with other PTMs.

### 4.7. Palmitoylation

Protein S-palmitoylation, a classical lipid modification process, describes the reversible conjugation of the fatty acid palmitate to the cysteine residues of target proteins. Emerging evidence indicates that palmitoylation is a key regulatory mechanism in the STING pathway [270]. In particular, palmitoylated STING is crucial for its assembly into multimeric complexes and for the recruitment of downstream signaling proteins in the Golgi apparatus [271]. The DHHC family comprises a group of proteins associated with palmitoylation. Activated STING translocates to the Golgi apparatus, where the DHHC family members DHHC3, DHHC7, and DHHC15 act as palmitoyltransferases, covalently modifying STING at C88 and C91 [272]. This palmitoylation reaction is effectively antagonized by the STING C88/91S mutant or the palmitoylation inhibitor 2-bromopalmitate (2-BP), which could be a potential feedback regulator that modulates STING signaling [271]. These results demonstrate that STING undergoes palmitoylation in the Golgi apparatus and that this PTM is indispensable for STING signaling.

### 4.8. Redox and Carbonylation

In addition to the unique nucleophilic nature of cysteine residues, the redox sites on their structures also confer different modifying functions, which realizes the fine regulation of many life activities. Recent studies have verified that oxidative PTMs involving cysteine are intimately linked to cGAS-STING signaling. Sustained cellular reactive oxygen species (ROS) during infection leads to the oxidation of STING at C64 and C148 and the formation of intermolecular disulfide bonds, which are sufficient to reduce the ability of STING to activate IFN-β expression [273,274]. In contrast, cytoglobin may protect STING from oxidation by scavenging ROS to promote IFN-β secretion in chronic liver disease [274]. 

Carbonylation, an irreversible oxidative protein modification, functions as a deleterious inhibitor of cGAS-STING signal transduction. For example, glutathione peroxidase 4 (GPX4) facilitates STING signaling by maintaining lipid redox homeostasis. Mechanistically, GPX4 inactivation during viral infection increased the cellular levels of the lipid peroxidation metabolite 4-HNE, which promoted STING to undergo carbonylation modification at C88, thus blocking subsequent translocation [275]. Additionally, as mentioned earlier, the S-nitroalkylation of STING is also dependent on the C88 site. Therefore, competition between these PTMs at the same residue is crucial for the delicate regulation of STING activity.

### 4.9. Caspase-Mediated Cleavage

Caspases are an evolutionarily conserved family of cysteine-dependent proteases that are actively involved in the execution of cell death, inflammation, and innate immunity [276]. Recent studies have revealed that the caspase family can cooperate with the cGAS-STING signaling pathway to form complex crosstalk. Yet, most caspase family members target key junctions of the cGAS-STING signaling pathway for negative regulation, thereby suppressing innate immunity. For example, during inflammasome activation, caspase-1 leads to cGAS inactivation and reduces cGAMP levels by recruiting and cleaving cGAS at D140/157, ultimately repressing type I IFN production [277]. This experiment further revealed that cGAS may also be cleaved by caspase-4, 5, and 11 in a different manner than caspase-1 under non-classical inflammasome activation conditions [277]. Collectively, these data support the conclusion that all inflammatory caspases are capable of cleaving cGAS; however, the specific mechanism requires further investigation. Another study reported that caspase-3 is also involved in cGAS cleavage. Upon DNA or RNA virus infection, activated caspase-3 strongly cleaves cGAS, mitochondrial antiviral signaling (MAVS), and IRF3 in DNA or RNA virus-infected cells, effectively avoiding cytokine overproduction [278]. An intriguing finding was that caspase-7 is only involved in the inactivation of cGAS in mouse cells, indicating the existence of a species-dependent immune mechanism. There is increasing evidence that mitochondrial apoptosis is associated with cGAS-STING signaling. As previously mentioned, mtDNA is released during apoptosis, triggering cGAS/STING-dependent type I IFN production. Instead, activated caspase-9 attenuates this response [279,280]. This conclusion is supported by the observation that the genetic deletion of caspase-9, caspase-3/7, or Apaf-1 leads to type I IFN production by dying cells [280]. Therefore, mutual coordination between the mitochondria-caspase-cGAS/STING signaling pathway plays a crucial role in regulating innate immune responses.

### 4.10. Protein–Protein Interaction

The chemical space and functional repertoire of proteins have been dramatically expanded by PTM of various amino acid residues [281]. In addition, the cGAS-STING signaling pathway is regulated by a variety of unknown mechanisms (Table 2), including protein–protein physical binding or blocking of the formation of activation complexes, which affect the enzymatic activity, stability, DNA binding, and translocation of cGAS-STING signaling. These studies highlight the critical role of disordered PTMs in the initiation, progression, and outcomes of various diseases. Based on these known and possibly additional unknown PTMs, the structures of cGAS/STING-related pathway proteins have become more complex, their functions enhanced, their regulation more refined, and their effects more pronounced.

## 5. Treatment with PTMs

In recent years, the cGAS-STING pathway has gained clinical importance owing to its great potential for type I IFN production and T cell priming. Several therapeutic strategies targeting the cGAS-STING pathway have been developed and tested using preclinical models [300,301,302]. In particular, the emerging understanding of the different biological mechanisms of PTMs targeting this pathway has also facilitated the development of novel therapeutic strategies. Nitrofuran derivatives, such as C-176, C-178, and H-151, have been identified to robustly antagonize STING palmitization by covalently binding to STING at Cys91 [271]. Moreover, unsaturated nitro-fatty acids (NO2-FAs) as anti-inflammatory agents have the ability to engage STING and affect its palmitoylation [303]. More strikingly, aspirin, a non-steroidal anti-inflammatory drug, has been demonstrated to play a broader role by directly modulating cGAS acetylation and impairing cGAS-mediated immune responses. In particular, the effective dose of aspirin required to inhibit cGAS in this experiment fell far below the upper limit of its use in humans [262]. This evidence provides a rationale for targeting the PTM pathway to prevent STING-induced autoimmune diseases. Unfortunately, these antagonists demonstrated low experimental cell activity and augmented cGAS-STING signaling only in mice but not in humans, which may account for the inability to relieve autoimmune symptoms in patients in clinical trials [304]. Together, the development of new STING agonists necessitates the results of the interaction and activation of human STING before clinical trials.

## 6. Conclusions and Future Directions

Since the discovery of the cGAS-cGAMP-STING pathway, a series of biochemical, structural, and genetic studies has established the basic framework and mechanism of this DNA-sensing pathway, although cGAS and STING in some species have been inactivated by mutations and premature stop codons [305]. Nonetheless, much remains to be learned regarding the PTM regulatory mechanisms of this pathway and the versatility of different levels of PTM crosstalk in pathophysiological processes. 

The discovery and characterization of the cGAS-STING pathway provides a novel framework for understanding the regulatory capacity of dsDNA immune recognition. Under normal physiological conditions, cGAS can recognize and bind to the invading pathogen DNA, triggering an innate immune reaction. In contrast, aberrant activation of the cGAS-STING pathway by endogenous DNA may also result in autoimmune and inflammatory diseases. Furthermore, the activation of the STING protein as a bridge between innate and adaptive immunity can effectively inhibit tumor progression. Consequently, the regulation of the cGAS-STING pathway and the expression of type I IFN and related inflammatory factors are of great significance for alleviating autoimmune diseases and intervening in the progression of malignant tumors.

As promising as this field is, there are some issues that require additional attention or solutions. Currently, experimental studies on cGAS-STING-related PTMs have mainly focused on antiviral immune responses. However, a covalent STING palmitoylation inhibitor was recently identified to attenuate metastasis during late-stage cancer therapeutic interventions [271]. Therefore, gaining an in-depth understanding of the PTM regulatory network of the cGAS-STING signaling axis would be an exciting advance in the fields of cancer immunology and clinical therapy. Future resources need to be allocated to the investigation of PTM sites in tumor cells to accelerate the understanding of the complex interactions between PTM regulation and the tumor microenvironment, and to develop improved therapeutic options for patients with cancer.

In summary, the regulation of the cGAS-STING pathway by numerous PTMs is a highly relevant and productive area of research that will undoubtedly deliver exciting discoveries in the coming years.

## Figures and Tables

**Table 1:** Viral Regulators of the cGAS-STING Pathway and proposed mechanisms. Abbreviations: VC: viral clearance; VE: viral escaping; HSV-1: Herpes simplex virus 1; DUB: deubiquitinase; KSHV: Kaposi’s sarcoma-associated herpesvirus; CBP: CREB-binding protein; MHV68: Murine gammaherpesvirus 68; VZV: Varicella-zoster virus; MDV: Marek’s disease virus; EBV: Epstein–Barr virus; LMP1: Latent membrane protein 1; HPV: Human papilloma virus; HBV: Hepatitis B virus; HCMV: Human cytomegalovirus; VACV: Vaccinia Virus; DNA-PK: DNA-dependent protein kinase; ECTV: Ectromelia virus; ASFV: African swine fever virus; HIV: Human immunodeficiency virus; DENV: Dengue virus; HCV: Hepatitis C virus; PEDV: Porcine epidemic diarrhea virus; TGEV: Tansmissible gastroenteritis virus; HCoV-NL63: Human coronavirus NL63; CHIKV: Chikungunya virus; WNV: West Nile virus; MNV: Murine norovirus; ZIKV: Zika virus; MeV: Measles virus; NiV: Nipah virus; SeV: Vesicular stomatitis virus; SINV: Sindbis virus; EMCV: Encephalomyocarditis virus; IAV: Influenza A virus; PRRSV: Porcine reproductive and respiratory syndrome virus; SFTSV: Severe fever with thrombocytopenia syndrome bunyavirus; T3D: Type 3 Dearing strain of reovirus.

Virus Types	Proposed Mechanism	Reference
**DNA Viruses**		
HSV-1	VC: releases extracellular vesicles; STING interaction; stabilizes STING in HEp-2 cells; enhances cGAMP levels; STING-mediated non-canonical autophagy	[27,50,51,52,53]
	VE: dampens NF-κB activation; degrades cGAS mRNA; binds to STING and TBK1; restrains cGAS catalyze activity; the deubiquitinase (DUB) activity; mediates cGAS deamidation; inhibits cGAS-DNA phase separation; obstructs TBK1 dimerization; induces TBK1 proteasomal degradation; interacts with karyopherin	[53,54,55,56,57,58,59,60,61,62]
KSHV	VC: detection by the cGAS	[16]
	VE: inhibits cGAS activity and production of cGAMP; blocks binding of cGAS to DNA; interacts with STING; inhibits IRF3 interaction with CBP; competes with IRF3 for IFNβ promoter binding	[63,64,65,66,67]
MHV68	VC: IFN-mediated antiviral pathways	[68]
	VE: DNA binding; DUB-dependent inhibition of STING; interacts with TBK1; blocks IRF3 and CBP interaction	[64,69,70,71]
VZV	VE: restricts cGAS-DNA phase separation; interacts with IRF3; prevents IRF3 phosphorylation at S396; inhibits IκBa ubiquitination	[60,72,73,74]
MDV	VE: hampers the combination of IRF7 and TBK1 with STING; impedes IRF7 phosphorylation and nuclear translocation	[75,76]
EBV	VE: regulation of LMP1; restricts cGAS-DNA phase separation	[60,77]
HPV	VC: binds to DNA virus	[78]
	VE: vesicular trafficking; degradation of STING; translation inhibition; STING interaction	[79,80,81,82]
Adenovirus	VC: detection by the cGAS	[83]
	VE: STING interaction	[82]
HBV	VC: detection by the cGAS and STING;	[84]
	VE: bounds to STING and attenuates K63-linked polyubiquitination of STING	[85]
HCMV	VC: induces cGAS; binds to IFI16 and relocalizes IFI16 to the cytoplasm	[86,87]
	VE: removes K63 ubiquitination of STING; inhibits the translocation of STING and impairs the recruitment of TBK1 and IRF3; induces STING degradation and inhibits cGAMP-mediated IFN-β induction; cGAS interaction; disrupts STING oligomerization and STING-TBK1 association; protein S-nitrosylation; hinders STAT1 phosphorylation	[88,89,90,91,92,93,94]
VACV	VC: activation of NF-κB and IRF3	[95,96]
	VE: selective 2’3’cGAMP degradation; interaction of STING with sulfated glycosaminoglycans; suppresses STING phosphorylation and dimerization; mTOR-dependent cGAS degradation; preventes cGAMP spread; blocks the activation of TBK1 and IKKε; binds to the Ku70-Ku80 complex and blocks DNA sensing by DNA-PK in fibroblasts	[97,98,99,100,101,102]
ECTV	VC: detection by the cGAS-STING pathway; induces the Phosphorylation of TBK1 and IRF3	[95]
	VE: suppresses activation of STING and IRF3	[99]
	VE: activation of IRF7 and NF-κB signaling	[103]
ASFV	VC: induces STING phosphorylation and trafficking	[104]
	VE: suppresses TBK1 phosphorylation and IKKβ; impairs STING activation; IKKε interaction; the autophagy-mediated lysosomal degradation of TBK1; suppression of NF-κB and IRF3 activation	[104,105,106,107,108]
**Lentiviruses**		
HIV	VC: cGAS interaction; recognizes cDNA (ssDNA) reverse-transcribed from HIV-1 virus; intercellular transfer of cGAMP; detects/disrupts nuclear viral capsid	[47,109,110,111,112]
	VE: dampens IRF3 and NF-kB nuclear translocation	[62,113]
**RNA viruses**		
DENV	VC: modulates the overall refractoriness of cells; induces mtDNA release; interacts with RIG-I and MAVS	[28,68,114]
	VE: binds to and cleaves STING; targets cGAS for degradation	[115,116]
HCV	VC: addition of cGAMP or STING inhibits viral replication	[117]
	VE: interacts with STING to disrupt the interaction of STING with TBK1 or MAVS and downstream IFN signalling; suppresses STING accumulation	[117,118,119]
SARS-CoV-2	VC: activates of STING; cell fusion; triggers the cGAS/STING axis	[120,121,122,123]
	VE: cleaves ubiquitin and ISG15; STING interaction; disrupts dimerization and K63-linked polyubiquitination of STING; interacts with TBK1 and impedes the phosphorylation and nuclear translocation of IRF3; interaction with the STING-TRAF3-TBK1 complex; the deubiquitinating activity	[120,124,125,126,127]
PEDV	VE: interacts with STING and represses K63-linked polyubiquitination of STING	[128]
TGEV	VE: deubiquitination of STING	[129]
HCoV-NL63	VE: binds to STING to disrupt its dimerisation/ubiquitination and downstream IFN production; DUB activity	[127,130]
CHIKV	VC: restricts CHIKV replication	[131,132]
	VE: induces cGAS degradation	[131]
WNV	VC: modulates T cell responses and T cell frequencies; restricts WNV infection	[68,133]
	VE: cleaves hSTING; inhibits the phosphorylation of TBK1 and IRF3	[134,135]
MNV	VC: limits MNV infection; drives cytosolic DNA accumulation and cGAS/STING activation	[136]
ZIKV	VC: STING interaction; induces antiviral autophagy	[137,138,139]
	VE: promotes the cleavage of cGAS; cleaves STING	[134,140]
MeV	VC: induces phosphorylation and ubiquitination of STING; induces mtDNA release	[141,142]
NiV	VC: induces phosphorylation and ubiquitination of STING	[141]
SeV	VC: translation inhibition; anti-SeV activity; induces STING expression	[143,144,145,146]
VSV	VC: translation promotion	[143]
	VE: reduces the ratio of full-length wt hSTING/truncated STING isoforms	[147]
SINV	VC: translation inhibition	[143]
EMCV	VC: stimulates mtDNA release and consequent cGAS activation; restricts EMCV replication	[144,148]
IAV	VC: membrane fusion and STING interaction; monoubiquitination of cGAS; triggers mtDNA release and binds to mtDNA; inhibits IAV replication	[146,148,149,150]
PRRSV	VC: suppress PRRSV replication	[150]
SFTSV	VC: Cytosolic SAFA senses viral RNA and activates STING antiviral signal	[151]
T3D	VC: translation inhibition	[143]

**Table 2:** Regulation of the cGAS-STING signaling by Direct Physical Interaction. Abbreviations: G3BP1: GTPase-activating protein-binding proteins 1; PQBP1: polyglutamine binding protein 1; ZCCHC3: zinc finger CCHC-type containing 3; OASL: oligoadenylate synthetases-like; SAR1A: secretion-associated and RAS-related; DDX41: DEAD-box helicase 41; TMED2: transmembrane emp24 protein transport domain containing 2; iRhom2: inactive rhomboid protein 2; STIM1: stromal interaction molecule 1; TOLLIP: toll-interacting protein; IFI16: interferon-inducible protein 16; NLRX1: NOD-like receptor (NLR) protein; NLRC3: NOD-like receptor family CARD domain containing 3; ISG56: IFN-stimulated gene 56; ZDHHC1: zinc finger DHHC-type containing 1; TMEM203: trans-membrane 203.

Target Protein	Factors	Functions	PTM Effects	Reference
cGAS	G3BP1	Promoting the formation of large cGAS complexes	Efficient activation of cGAS	[282]
cGAS	PQBP1	Increasing DNA-binding affinity	Initiates an IRF3-dependent innate response	[283]
cGAS	ZCCHC3	Enhancing the binding of cGAS to dsDNA	Acts as a general co-sensor of cGAS	[284]
cGAS	β-arrestin 2	Enhancing the DNA-binding ability of cGAS	Promotes IFN-β signaling	[285]
cGAS	Beclin-1	Regulating cGAMP production and autophagy	Balances anti-microbial immune responses	[286]
cGAS	OASL	Binding to cGAS and inhibiting cGAMP synthesis	Inhibits cGAS-mediated IFN induction	[287]
STING	SAR1A and SEC24C	Blocking STING puncta formation induced by cGAMP	Affects STING trafficking and signalling	[27]
STING	DDX41	Interacting with DNA and STING	Activates protein kinases, TBK1, NF-κB and IRF3	[288,289]
STING	TMED2	Reinforcing STING dimerization and facilitating its trafficking	Potentiates cellular IFN responses to DNA viruses	[290]
STING	iRhom2	Recruiting the translocon-associated protein TRAPβ	Facilitates the trafficking of STING	[240]
STING	STIM1	Retaining the signaling adaptor STING at the ER	Regulates the type I interferon response	[291]
STING	TOLLIP	Interacting with STING N-terminus	Stabilizes resting-state STING protein levels	[292]
STING/TBK1	IFI16	Promoting production and function of cGAMP	Regulates the activation of STING and the recruitment of TBK1	[293,294]
STING/TBK1	NLRX1	Associating with STING to disrupt STING-TBK1 interaction	Inhibits innate immunity and facilitates viral spread	[295]
STING/TBK1	NLRC3	Impeding the interaction between STING and TBK1	Negatively regulates the STING-mediated DNA-sensing pathway	[296]
STING/TBK1	ISG56	Disrupting the interactions between STING and TBK1,	Inhibits antiviral signaling	[297]
STING/TBK1	ZDHHC1	Mediating dimerization/aggregation of STING and recruitment of TBK1 and IRF3	Positively regulates the innate immune response against DNA viruses	[298]
STING/TBK1	TMEM203	Forming a functional signaling complex with STING	Promotes the TBK1-IRF3-IFN activation	[299]

## Abbreviations

PRRs: pattern-recognition receptors; cGAS: Cyclic GMP-AMP synthase; STING: stimulator of interferon genes; PTMs: post-translational modification; IFNs: type I interferons; NTase: nucleotidyltransferase; dsDNA: double-stranded DNA; 2′,3′-cGAMP: 2′,3′-cyclic GMP-AMP; CDN: cyclic dinucleotide; ER: endoplasmic reticulum; CBD: CDN-binding domain; CTT: C-terminal tail; TBK1: TANK-binding kinase 1; IRF3: IFN regulatory factor 3; ERGIC: ER–Golgi intermediate compartment; COPII: complex II; ARF: ADP-ribosylation factor; ISGs: IFN-stimulated genes; IKK: IκB kinase; NF-κB: nuclear factor-κB; HR: homologous recombination; DCs: dendric cells; CTL: cytotoxic T lymphocyte; Treg: regulatory T; PDE4: phosphodiesterase 4; CAMP: cyclic adenosine monophosphate; NK: natural killer; SASP: senescence-associated secretory phenotype; Bcl-2: B-cell lymphoma 2; BAX: Bcl-2 related X protein; BAK: Bcl-2 homologous antagonist/killer; Syk: splenic tyrosine kinase; SLE: systemic lupus erythematosus; AGS: Aicardi–Goutières syndrome; SAVI: STING-associated vasculopathy with onset in infancy; DNases: Deoxyribonucleases; TREX1: three-prime repair exonuclease 1; AKT1: AKT serine/threonine kinase 1; PKB: protein kinase B; HSV: herpes simplex virus; CDK1: cyclin-dependent kinase 1; PP1: protein phosphatase 1; PSP: protein serine/threonine phosphatase; PPM1A: protein phosphatase Mg2+/Mn2+- dependent 1A; S6K1: S6 kinase 1; PTPN: tyrosine-protein phosphatase nonreceptor type; DYRK2: Dual-specificity tyrosine-(Y)-phosphorylation-regulated kinase 2; DNA-PK: DNA-dependent protein kinase; PP2A: protein phosphatase 2A; Ub: ubiquitin; DUBs: deubiquitinases; MUL1: mitochondrial E3 ubiquitin protein ligase 1; MYSM1: Myb-like, SWIRM, and MPN domains 1; AMFR: autocrine motility factor receptor; iRhom2: inactive rhomboid protein 2; EBV: Epstein–Barr virus; HBV: Hepatitis B virus; DAPK3: death-associated protein kinase 3; SUMO: small ubiquitin-like modifier; TRIM: tripartite motif; SENP: sentrin-specific protease; UBLs: ubiquitin-like proteins; HDAC-3: histone deacetylase 3; PRMT5: protein arginine methyltransferase 5; TTLL: tubulin tyrosine ligase-like; Asn: asparagine; Gln: glutamine; Asp: aspartic acid; Glu: glutamic acid; dsRNA: double-stranded RNA; 2-BP: 2-bromopalmitate; ROS: reactive oxygen species; GPX4: glutathione peroxidase 4; MAVS: mitochondrial antiviral signaling; NO2-FAs: nitro-fatty acids.
